# Bridging the gap in young adult care: the educational value of implementing Student Health Centers in primary care training

**DOI:** 10.1017/S1463423626101637

**Published:** 2026-08-12

**Authors:** Tumay Sadikoglu, Duygu Altiparmak, Helen Shields

**Affiliations:** 1 Yeditepe University School of Medicine: Yeditepe Universitesi Tip Fakultesi, Turkey; 2 Harvard Medical School Department of Medicine, USA

**Keywords:** family medicine, primary care training, Student Health Center, undergraduate medical education

## Abstract

**Aim:**

This study aims to evaluate the feasibility and educational impact of utilizing SHCs as primary care training sites in undergraduate medical education, with the goal of enhancing clinical competencies in young adult healthcare.

**Background:**

Family Medicine (FM) is essential for integrating community-based health services into medical education. In Turkey, final-year medical students complete a one-month rotation at Family Health Centers (FHCs). However, young adults often utilize Student Health Centers (SHCs) on university campuses, resulting in a gap in students’ experience with this age group. Addressing this, our programme initiated the inclusion of SHCs in the FM internship rotation for the 2024–2025 academic year.

**Methods:**

A mixed-methodology study was conducted in two phases. A retrospective analysis was performed on medical records of patients aged 18–25 who attended the SHC over a one-month period. A prospective 9-item online survey assessed the educational impact on final-year medical students following their FM internship, which included the SHC rotation. Descriptive and qualitative statistical analyses were performed to summarize the survey and clinical data.

**Findings:**

A total of 1228 patients were seen at the SHC, mirroring FHC trends. All 87 interns surveyed rated the FM programme initiative positively, with 94.1% highly satisfied and 85.9% reporting improved primary care skills. Qualitative feedback highlighted the value of practical experience and called for longer rotations.

**Conclusion:**

Integrating SHC rotations into undergraduate medical education is both feasible and beneficial, providing relevant primary care experience for medical students and enhancing training in the care of young adult populations.

## Introduction

Family medicine (FM), as a cornerstone of primary care, occupies a unique and essential position in the contemporary landscape of medical education. Its scope and philosophy emphasize comprehensive, continuous, and coordinated care, making it a prime candidate for adapting medical school curricula to better align with community-based health services and real-world patient needs. Over the past decade, medical educators and policymakers have increasingly recognized that the dominant hospital-centric model of clinical training – while valuable – does not fully prepare future physicians for the realities of outpatient, ambulatory, and community-oriented care, which constitute the overwhelming majority of patient encounters in the healthcare system. The transition from theoretical learning to practical application is most effectively achieved when students are exposed to the environments where patients live, work, and seek care for both acute and chronic conditions.

FM often provides students with their earliest exposure to direct patient care, particularly during their preclinical years. This early introduction is not only foundational for developing clinical reasoning and core procedural skills but is pivotal in shaping attitudes toward the doctor-patient relationship, fostering empathy, and highlighting the importance of continuity and patient-centeredness in medical practice. Exposure to FM at this critical stage helps medical students appreciate the broader social, psychological, and preventive dimensions of care, which are often underemphasized in more specialized or inpatient settings.

As students’ progress into their clinical years, the emphasis in FM education shifts toward more structured, immersive experiences, including block rotations, longitudinal clerkships, and multidisciplinary collaborations. These typically involve integration with other specialties such as paediatrics, internal medicine, and geriatrics, ensuring that students gain a holistic understanding of the patient’s journey across the lifespan. The vertical integration of FM throughout the curriculum has been shown to enhance students’ appreciation for the breadth and depth of primary care, as well as the versatility required of family physicians in addressing a variety of health problems and needs (Turkeshi *et al*., 2015).

In Turkey, undergraduate medical education is designed to provide a comprehensive overview of clinical practice through a series of internship rotations in major specialties, including Obstetrics and Gynecology, Emergency Medicine, General Surgery, Psychiatry, Public Health, and Family Medicine. The structure of these rotations, particularly the one-month mandatory placement in Family Health Centers (FHCs) during the sixth and final year, reflects a national commitment to ensuring that all graduates are familiar with the principles and practice of primary care. This placement, usually arranged with approval from the local District Health Directorate, is intended to immerse students in the realities of primary care provision outside the traditional university setting. By the end of this rotation, students are expected to demonstrate competence in core clinical and communication skills, fulfilling both academic and health system expectations.

FHCs, serving as the bedrock of primary healthcare delivery in Turkey, provide continuous, comprehensive preventive, diagnostic, therapeutic, and rehabilitative services to individuals of all ages and backgrounds. Their remit extends beyond episodic care to include health education, promotion, prevention, maternal and child health, family planning, and the organization of screening programmes tailored to specific risk profiles (Republic of Turkiye Ministry of Health, 2024). Such programmes cater to diverse groups, including children, adolescents, adults, the elderly, and those facing particular health challenges such as chronic disease or pregnancy. When patient needs exceed the capacity of the FHC, referrals are coordinated to ensure seamless transitions to higher-level or specialized care, including home-based services. In addition, FHCs are responsible for preparing a variety of health reports, referral and prescription documentation and fulfilling legal obligations as stipulated in national healthcare legislation (Republic of Turkiye Ministry of Health, 2024). This comprehensive, patient-centred approach is designed to ensure equitable, high-quality care for all, regardless of demographic or socioeconomic factors.

Internationally, the centrality of primary healthcare has been repeatedly validated as a driver of improved population health, reduced disparities, and more equitable access to necessary services (Starfield *et al*., 2005; World Health Organization, 2013). Within this context, Student Health Centers (SHCs) on university campuses have emerged as robust, dynamic primary care sites, mirroring many of the functions and responsibilities of FHCs (Hicks and Dinkel, 2016). SHCs go beyond treating acute illnesses or minor injuries; they are active in health education, promotion, preventive screenings, immunizations, and counselling, especially on issues pertinent to young adults, such as sexual health and mental well-being.

Despite the potential for continuity of care in the FM model, young adults – particularly university students – tend to underutilize their designated family physicians, often favouring the convenience and accessibility of SHCs (Mac and Ozturk, 2018; Bilgili *et al*., 2023). This trend has multiple drivers, including the transitional and often transient nature of student life, a strong desire for confidential and age-appropriate services, and the perception that SHCs are better equipped to meet the unique needs of young adults. As a result, young adults frequently seek care during times of acute stress, illness, or crisis rather than maintaining an ongoing relationship with a family physician. This episodic approach to healthcare can disrupt continuity and deprive both students and their providers of opportunities to develop durable therapeutic alliances.

A recent Turkish study identified clinical complaints, prescription requests, and preventive health services as the leading reasons for visits to a family physician. The most common diagnoses and treatments included essential hypertension, musculoskeletal disorders, sore throat, cough, dyspepsia, and headache (Bilgili *et al*., 2023). Comparatively, the mean age of regular FHC patients is 35.8 years in Canada (Ryan *et al*., 2011) and 39.9 years in Turkey, with prescription frequency highest among those aged 45 and older (Bilgili *et al*., 2023). Another large-scale Turkish study reported that while 27.8% of primary care patients were aged 18–29, and 45.4% between 30 and 50, young adults (18–25 years) still make up a significant portion of the wider population – 15.2% according to recent national statistics (Turkish Statistical Institute, 2022). Despite their numbers, young adults are underrepresented in FHC visits, often choosing SHCs or emergency departments over scheduled appointments with a family physician, especially during periods of heightened stress or acute illness.

Furthermore, frequent transitions – such as moving between districts when enrolling in or graduating from university – add complexity to young adult care. Many students register with a family physician in a district where they do not permanently reside, fragmenting their healthcare records and diminishing continuity (Willis and McDonagh, 2018; Young Adults Joint Strategic Needs Assessment Report, 2025). The transient and mobile nature of this population presents unique challenges for traditional FHC-based training, which may not offer sufficient contact with young adults for meaningful educational encounters.

These considerations lead us to hypothesize that SHCs are not only well positioned to serve the healthcare needs of young adults but can also function as effective, high-value clinical training environments for medical students. The distinctive patient mix, prevalence of acute and preventive presentations, and opportunity for independent clinical decision-making make SHCs a valuable addition to traditional FM rotations.

Our study aims to evaluate the feasibility and educational benefits of including SHC rotations in undergraduate medical training, culminating in the integration of this model into the 2024–2025 FM internship curriculum of our medical school as a pilot initiative.

## Methods

### Research design

This mixed-methodology study was conducted in two phases, combining retrospective and prospective approaches to evaluate both the clinical and educational dimensions of SHC-based FM training.

The first phase entailed a retrospective review of medical records for university students aged 18–25 attending the SHC between October 25 and November 25, 2023. Data were extracted on age, gender, and reasons for presentation, providing a descriptive overview of the clinical spectrum and patient flow at the SHC. The analysis was conducted using SPSS version 21.0, with results expressed as frequencies, percentages, and means. This phase established the baseline characteristics of the SHC patient cohort, enabling comparison with more traditional FHC populations.

The second phase prospectively assessed the educational impact of a FM internship that included a structured SHC rotation. This rotation was implemented from September 2024 to June 2025, involving all sixth-year medical students assigned to the FM internship during this period. Upon completion, students were invited to complete a 9-item online survey via SurveyMonkey, evaluating satisfaction, perceived learning gains, and suggestions for improvement. Eight survey items used a 5-point Likert scale, and one allowed for open-ended comments (Appendix A). The survey instrument was developed based on a comprehensive literature review and primary care educational standards. Content validity was evaluated by a panel of three experts in FM and medical education, and items were revised based on their feedback. A pilot test was conducted with a small cohort of medical students (*n* = 10, excluded from the final analysis) to assess clarity, readability, and feasibility. Internal consistency of the Likert-scale items was evaluated using Cronbach’s alpha, yielding a coefficient of 0.84, indicating high reliability.

All participants provided informed consent, and data confidentiality was maintained throughout.

### Statistical analysis

Statistical analyses were performed using SPSS Statistics for Windows, version 26.0 (IBM Corp., Armonk, NY, USA), with a significance level of *α* = 0.05. Descriptive statistics were used to summarize the data and are presented as frequencies, percentages, means, and standard deviations, as appropriate. Survey responses were analysed descriptively to evaluate students’ perceptions of the educational value of the Student Health Center rotation.

QuillBot AI (version 2.0.0.0) was utilized as an assistive tool during the qualitative data analysis phase of this study. Specifically, QuillBot AI supported the thematic coding and organization of open-ended survey responses by aiding in the identification of recurring patterns and themes. All outputs generated by this programme were reviewed and validated independently by two human researchers to ensure accuracy, reliability, and alignment with established qualitative research standards. The use of QuillBot AI adhered to ethical guidelines, with all data anonymized prior to analysis. Qualitative responses were coded thematically to identify recurrent themes and unique perspectives. The data obtained from open-ended survey questions underwent thematic analysis. The analysis was conducted independently by two researchers to ensure investigator triangulation. First, the researchers familiarized themselves with the data through repeated reading. Open coding was performed to generate initial codes, which were then grouped into potential sub-themes and main themes. Discrepancies in coding and theme assignment were resolved through discussion and consensus with a third senior researcher. To ensure trustworthiness and reliability, member checking was performed with a subset of participants, and an audit trail was maintained throughout the analysis.

## Results

A total of 1,228 students (mean age: 21 ± 3.4 years; 738 female, 490 male) attended the SHC during the one-month period. The majority of visits were for first aid (24.4%), health reports (fitness certificates, vaccination records), and respiratory complaints, chiefly sore throat. Other common reasons included cough, headache, musculoskeletal discomfort, and gastrointestinal symptoms. Urological issues were rare (0.1%). These presentations closely mirrored those seen at FHCs, underscoring the representativeness of the SHC as a primary care training site.

Patterns of SHC utilization reflected the unique health-seeking behaviours of young adults, with notable surges at the start of academic terms and during exam periods, when stress-related and infectious symptoms were more prevalent. Services ranged from acute care for minor injuries and illnesses to preventive care (immunizations, screenings, health education), and referrals for specialist input as needed. This comprehensive scope of care is consistent with the expectations for family physicians, reinforcing the educational value of SHC-based rotations.

### Educational value of SHC rotation

**Figure 1.**
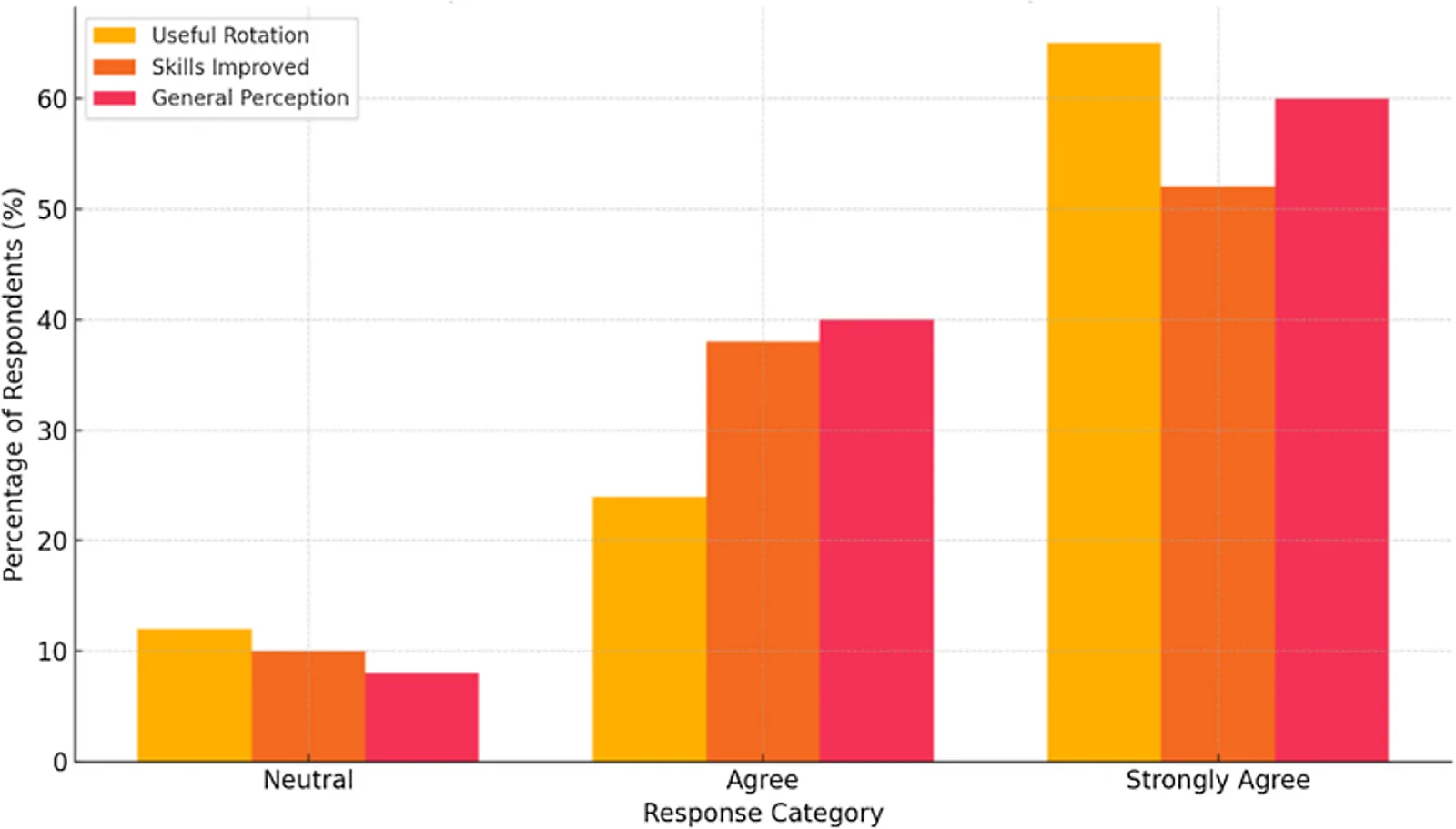
Overall assessment of the family medicine internship at the Student Health Center.

All 87 sixth-year medical students who completed the integrated FM internship participated in the post-rotation survey. Overall satisfaction was high, with 94.1% (*n* = 80) of students reporting a positive evaluation. Overall satisfaction was high, with 94.1% (*n* = 80) reporting a positive evaluation (Figure 1). Only 5.88% were neutral or unsure, and no negative responses were recorded.

The majority of students (89.4%, *n* = 76) agreed or strongly agreed that the SHC rotation enriched their understanding of primary care. The majority of students (89.4%, *n* = 76) agreed or strongly agreed that the SHC rotation enriched their understanding of primary care. Additionally, 85.9% (*n* = 73) felt that the rotation significantly enhanced their practical skills (Table 1), particularly through opportunities for hands-on procedures and management of the young adult patient population.

**Table 1.** Students’ perceptions regarding improvement in primary care skills following the Student Health Center rotation Table 1 long description.

Response option	*n* (%)
Strongly disagree	1 (1.1)
Disagree	3 (3.4)
Neutral	8 (9.2)
Agree	29 (33.3)
Strongly agree	44 (50.6)
**Total**	**85 (100.0)**

### Qualitative feedback

Seventeen students offered detailed narrative feedback, revealing several dominant themes. Examples of some statements of the students are quoted as follows:

- **Desire for Extended Rotations:** Many advocated for longer placements, believing more time would allow deeper practical learning.

‘I contend that the time in the SHC is insufficient. Prolonging this duration would enable us to acquire further practical experience.’

- **Need for Greater Clinical Diversity:** Some students wished for exposure to a wider variety of patient cases and conditions.

‘We would gain from encountering a broader spectrum of patients and conditions throughout our rotations.’’

- **Emphasis on Supervisor Feedback:** Students highlighted the value of regular, specific feedback in supporting reflection and professional growth.
- ‘Increased frequency and specificity of feedback from our supervisors would enhance our comprehension of our strengths and areas requiring improvement.’
- **Valuing Practical Experience:** The chance to perform real clinical procedures was repeatedly cited as the most instructive aspect.

‘Performing blood sampling and ECGs in the SHC constituted the most instructive aspect of the internship for me.’

In summary, qualitative responses demonstrated the value of hands-on learning, constructive feedback, and the desire for more prolonged and varied clinical exposure.

## Discussion

### Educational impact and student experience

The findings from this study underscore the value and feasibility of incorporating Student Health Centers into the undergraduate medical curriculum as primary care training environments. High satisfaction rates and strong positive feedback suggest that SHC rotations are not only acceptable but are perceived as highly beneficial by medical students. These rotations provide valuable opportunities to address common acute and preventive health issues relevant to young adults, develop procedural competencies, and appreciate the principles of FM in a real-world setting. Such experiences resonate with the established evidence that early, practical clinical exposure is critical for preparing students for the demands of primary care (Tayade and Latti, 2021).

The demographic focus of SHCs – predominantly young adults – offers unique teaching advantages. Students encounter a patient group that is often underrepresented in traditional FHCs but presents specific challenges and learning opportunities. These include mental health concerns, sexual and reproductive health counselling, preventive interventions, and the management of acute complaints in otherwise healthy individuals. This contrasts with the more chronic disease-oriented case mix commonly encountered in FHCs. By interacting with young, mobile, and often digitally literate patients, students also gain insights into the evolving expectations and preferences of future healthcare consumers.

### Alignment with global trends in medical education

Globally, there is growing recognition of the centrality of primary care in improving health outcomes, mitigating disparities, and optimizing resource allocation (Starfield *et al*., 2005; World Health Organization, 2013). International organizations and curricula increasingly emphasize early, longitudinal, and community-based experiences in primary care as vital components of medical education. The integration of SHC rotations aligns with these trends, offering a model that balances exposure to acute and preventive care, fosters interest in primary care careers, and supports workforce development.

Evidence from other settings supports the notion that experiential, community-based training enhances both skill acquisition and professional identity formation, increasing the likelihood that graduates will pursue careers in FM or other primary care specialties (Turkeshi *et al*., 2015). The SHC model thus serves not only educational goals but also broader health system objectives by attracting and retaining talent in critical areas.

### Comparative value: SHCs versus traditional FHCs

While FHCs remain the gold standard for primary care training in Turkey, this study demonstrates that SHCs provide a comparable environment in terms of patient case mix, procedural opportunities, and exposure to core primary care competencies. The similarities in clinical presentations further support the appropriateness of SHCs as a supplementary training site, particularly for skills related to acute care, health promotion, and the management of transitional life stages (Mac and Ozturk, 2018).

Furthermore, the organizational and resource constraints often encountered in SHCs encourage students to develop adaptability, creativity, and teamwork – skills that are increasingly essential in modern healthcare. The multidisciplinary nature of SHCs, which may include nurses, counsellors, and public health professionals, provides a rich context for interprofessional education.

The results of this study support the formal integration of SHC rotations into undergraduate FM curricula. Key considerations for curriculum design include ensuring sufficient duration, diversity of clinical exposure, structured supervision, and regular formative feedback. Incorporating objective structured clinical examinations (OSCEs) and performance-based assessments may further enhance the educational value of these rotations.

As digital health and telemedicine technologies become more prevalent, SHCs – serving a digitally native population – are ideal settings for training students in these modalities. This ensures that future physicians are prepared to meet the evolving needs and expectations of their patients, both in-person and remotely.

### Limitations and future research directions

Several limitations warrant consideration. This study was conducted at a single institution, and the relatively brief duration of the SHC rotation may limit external validity. The reliance on self-reported outcomes introduces the potential for response bias, and the absence of long-term follow-up precludes assessment of sustained impacts on clinical competence or career choice.

Future research should involve larger, multi-institutional samples and incorporate objective measures of skill acquisition and performance. Longitudinal studies tracking the career trajectories of graduates exposed to SHC training would provide valuable insights into the broader impact of this educational innovation. Qualitative studies involving SHC supervisors The inclusion of objective measures of performance, such as OSCEs, Mini-CEX, DOPS, or supervisor evaluations, would strengthen the conclusions, and patients could also enrich our understanding of the benefits and challenges of integrating students into these settings.

## Conclusion

Integrating Student Health Center rotations into undergraduate medical education is both feasible and highly valued by students. SHCs offer a distinctive and complementary environment for learning primary care, especially in areas relevant to young adults. Through careful curriculum design, adequate duration, and ongoing evaluation, SHCs can play a significant role in preparing the next generation of primary care physicians. This model enhances clinical and professional competence and supports the development of a workforce dedicated to excellence and innovation in primary care.

## Supporting information

10.1017/S1463423626101637.sm001 Sadikoglu et al. supplementary material Sadikoglu et al. supplementary material

## Data Availability

The data of the study is available upon request via an email sent to the corresponding author. Tumay Sadikoglu, MD: tumay.sadikoglu@yeditepe.edu.tr.

## Supplementary material

The supplementary material for this article can be found at https://doi.org/10.1017/S1463423626101637.

## Table 1. Long description

A table showing students’ perceptions regarding improvement in primary care skills following the Student Health Center rotation. The table has six rows and two columns. The columns are labeled ‘Response option’ and ‘n (%)’. The rows list different response options and their corresponding percentages and counts. The response options are ‘Strongly disagree’, ‘Disagree’, ‘Neutral’, ‘Agree’, ‘Strongly agree’, and ‘Total’. The values in the table are as follows: Row 1: Strongly disagree, 1 (1.1 percent). Row 2: Disagree, 3 (3.4 percent). Row 3: Neutral, 8 (9.2 percent). Row 4: Agree, 29 (33.3 percent). Row 5: Strongly agree, 44 (50.6 percent). Row 6: Total, 85 (100 percent). The boldface in the table highlights the majority response, which is ‘Strongly agree’ with 44 students (50.6 percent) indicating a significant positive perception of the rotation’s impact on their primary care skills.

Navigate back to Table 1..
