# Supplementary material for: Bridging the gap in young adult care: the educational value of implementing Student Health Centers in primary care training
Source: Prim Health Care Res Dev. 2026 Aug 12;27:e92. doi: 10.1017/S1463423626101637 (PMC13740156; doi:10.1017/S1463423626101637)
Supplement: Supplementary file 1 [file S1463423626101637sup001.docx]

Appendix 1

**FAMILY MEDICINE INTERNSHIP EVALUATION FORM**

Thank you for your contribution to the Family Medicine Internship evaluation form. Please rate the statements below according to your thoughts.

**1: strongly disagree 2: agree 3: neutral 4: agree 5: strongly agree.**

| **Statements** | **Strongly Disagree**  **(1)** | **Disagree**  **(2)** | **Neutral**  **(3)** | **Agree**  **(4)** | **Strongly Agree**  **(5)** |
| --- | --- | --- | --- | --- | --- |
| My general perception about family medicine internship is positive. |  |  |  |  |  |
| I had the opportunity to observe the practical application of family medicine principles in a clinical context. |  |  |  |  |  |
| What I experienced in practice was aligned with what I have been taught in theory. |  |  |  |  |  |
| The internship was helpful for understanding the principles of family medicine. |  |  |  |  |  |
| I am interested in becoming a family physician after this rotation. |  |  |  |  |  |
| The level of communication between the family physicians and the patients was mostly adequate. |  |  |  |  |  |
| My time spent at the student health center significantly improved my skills in primary care. |  |  |  |  |  |
| I believe, the inclusion of student health center rotations in family medicine training has been really useful. |  |  |  |  |  |

**What are your suggestions for improvement?**
